# Treating BCG-Induced Cystitis with Combined Chondroitin and Hyaluronic Acid Instillations in Bladder Cancer

**DOI:** 10.3390/jcm13072031

**Published:** 2024-03-31

**Authors:** Renate Pichler, Johannes Stäblein, Andrea Mari, Luca Afferi, David D’Andrea, Gautier Marcq, Francesco del Giudice, Francesco Soria, Jorge Caño-Velasco, José Daniel Subiela, Andrea Gallioli, Karl H. Tully, Keiichiro Mori, Achim Herms, Benjamin Pradere, Marco Moschini, Laura S. Mertens, Martin Thurnher

**Affiliations:** 1 Department of Urology, Comprehensive Cancer Center Innsbruck, Medical University of Innsbruck, 6020 Innsbruck, Austria; johannes.staeblein@tirol-kliniken.at; 2 Unit of Oncologic Minimally-Invasive Urology and Andrology, Department of Experimental and Clinical Medicine, Careggi Hospital, University of Florence, 50134 Florence, Italy; andreamari08@gmail.com; 3 Department of Urology, Luzerner Kantonsspital, 6000 Luzern, Switzerland; luca.afferi@gmail.com; 4 Department of Urology, Comprehensive Cancer Center, Medical University of Vienna, 1090 Vienna, Austriamorikeiichiro29@gmail.com (K.M.); 5 Department of Urology, Claude Huriez Hospital, CHU Lille, 59000 Lille, France; 6 Department of Maternal Infant and Urologic Sciences, Policlinico Umberto I Hospital, ‘Sapienza’ University of Rome, 00185 Rome, Italy; francesco.delgiudice@uniroma1.it; 7 Urology Division, Department of Surgical Sciences, University of Studies of Torino, 10124 Turin, Italy; francesco.soria@unito.it; 8 Department of Urology, Gregorio Marañón University Hospital, 28007 Madrid, Spain; jorcavel@gmail.com; 9 Department of Urology, Hospital Universitario Ramón y Cajal, IRYCIS, Universidad de Alcala, 28034 Madrid, Spain; jdsubiela@gmail.com; 10 Department of Urology, Fundació Puigvert, Autonomous University of Barcelona, 08193 Barcelona, Spain; andrea.gallioli@gmail.com; 11 Department of Urology and Neurourology, Marien Hospital Herne, Ruhr-University Bochum, 44625 Herne, Germany; karlh.tully@gmail.com; 12 Department of Urology, The Jikei University School of Medicine, Tokyo 105-8461, Japan; 13 Neuro-Urology Unit, Department of Urology, Medical University of Innsbruck, 6020 Innsbruck, Austria; achim.herms@tirol-kliniken.at; 14 Department of Urology, La Croix du Sud Hospital, 31130 Quint-Fonsegrives, France; benjaminpradere@gmail.com; 15 Department of Urology, IRCCS Ospedale San Raffaele, Vita-Salute San Raffaele University, 20132 Milan, Italy; marco.moschini87@gmail.com; 16 Department of Urology, The Netherlands Cancer Institute, Antoni van Leeuwenhoek Hospital, 1066 CX Amsterdam, The Netherlands; ls.mertens@gmail.com; 17 Immunotherapy Unit, Department of Urology, Medical University of Innsbruck, 6020 Innsbruck, Austria; martin.thurnher@i-med.ac.at

**Keywords:** BCG, bladder cancer, cystitis, hyaluronic acid, chondroitin, intravesical instillations

## Abstract

In non-muscle invasive bladder cancer, Bacillus Calmette–Guérin (BCG) responders benefit from strong Th1-type inflammatory and T cell responses mediating tumor rejection. However, the corresponding lack of anti-inflammatory Th2-type immunity impairs tissue repair in the bladder wall and facilitates the development of cystitis, causing urinary pain, urgency, incontinence, and frequency. Mechanistically, the leakage of the glycosaminoglycan (GAG) layer enables an influx of potassium ions, bacteria, and urine solutes towards the underlying bladder tissue, promoting chronic inflammation. Treatments directed towards re-establishing this mucopolysaccharide-based protective barrier are urgently needed. We discuss the pathomechanisms, as well as the therapeutic rationale of how chondroitin and hyaluronic acid instillations can reduce or prevent BCG-induced irritative bladder symptoms. Moreover, we present a case series of five patients with refractory BCG-induced cystitis successfully treated with combined chondroitin and hyaluronic acid instillations.

## 1. Introduction

Intravesical instillations with Bacillus Calmette–Guérin (BCG) are part of the success story of cancer immunotherapy for more than forty years [1]. BCG applications are still the standard adjuvant treatment in high-risk, non-muscle-invasive bladder cancer [2]. Repetitive intravesical BCG applications induce a local immune response that ultimately translates into an increased immune responsiveness termed trained immunity [3]. Consequently, intravesical BCG has not only local but also systemic effects.

BCG-induced cystitis is the most commonly reported local side effect of BCG treatment, and patients have the same symptoms as in non-BCG, bacterial urinary tract infections [4]. Irritative bladder symptoms are described in about 30–60% of cases, and patients complain of urgency, urinary frequency, nocturia, hematuria, and pain in the bladder region [4]. Interestingly, the rate of side effects showed no differences in age, BCG strains, or maintenance schedule [5,6,7]. The management of BCG-induced cystitis is clearly recommended in stages by current guidelines [2], including the discontinuation of the instillations and empirical antibiotic treatment, then anti-tuberculosis drugs with corticosteroids if the symptoms persist or, finally, a radical cystectomy in the case of contracted bladder or refractory symptoms. However, many patients show persistent symptoms which lead to a reduced quality of life (QoL) leading to the discontinuation of BCG treatment [2,8].

## 2. T Helper (Th) Cells in BCG-Induced Antitumor Effects and Bladder Inflammation and Repair

**Figure 1:**
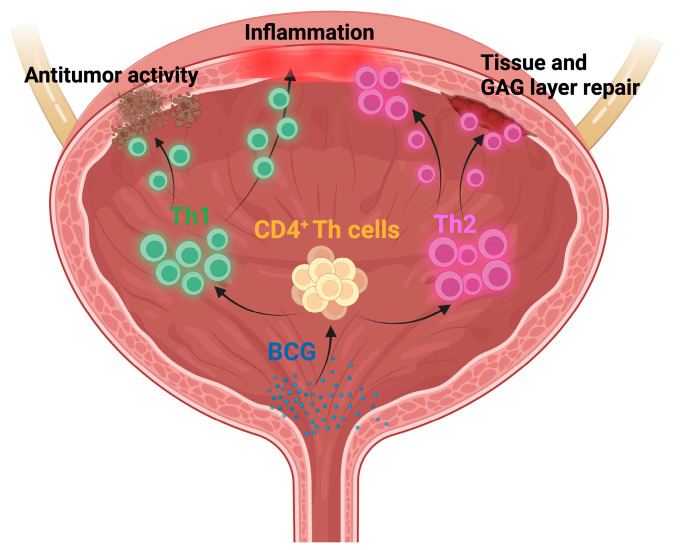
T helper (Th) cells in BCG-induced antitumor effects and bladder inflammation and repair. Intravesical application of BCG induces recruitment of CD4^+^ Th cells into the urothelium. A Th1-dominant bladder microenvironment is needed for an effective antitumor response. However, BCG-induced Th1-type inflammation also promotes cystitis. In contrast, Th2 T cells are required to repair the bladder wall and to restore the glycosaminglycan (GAG) layer [12].

BCG enters the bladder wall at the tumor resection site through complexes with fibronectin [9]. Then, antigen-presenting cells in the urothelium phagocytose the BCG and present BCG-derived antigens to CD4^+^ T helper (Th) cells, which produce either Th1-type or Th2-type cytokines [10]. A local inflammatory immune response with an influx of granulocytes and mononuclear cells and the potent production of pro-inflammatory Th1 cytokines is associated with the BCG response [11]. During bladder infections, CD4^+^ Th cells, cytotoxic T cells, and γδ T cells are recruited into the bladder to mediate antitumor immune responses. In contrast, the Th2 T cells mainly serve to repair the tissue damage in the bladder wall induced by bacterial infections and the Th1-type responses. Upon repair, the intact urothelium is able to restore the glycosaminoglycan (GAG) layer [12]. Thus, Th cell subsets play an essential role not only in BCG-induced antitumor activity but also in the subsequent bladder tissue repair (Figure 1).

## 3. Physiological Function of the GAG Layer and the Rationale of Treating BCG-Induced Cystitis with Intravesical Instillations of GAG Components

**Figure 2:**
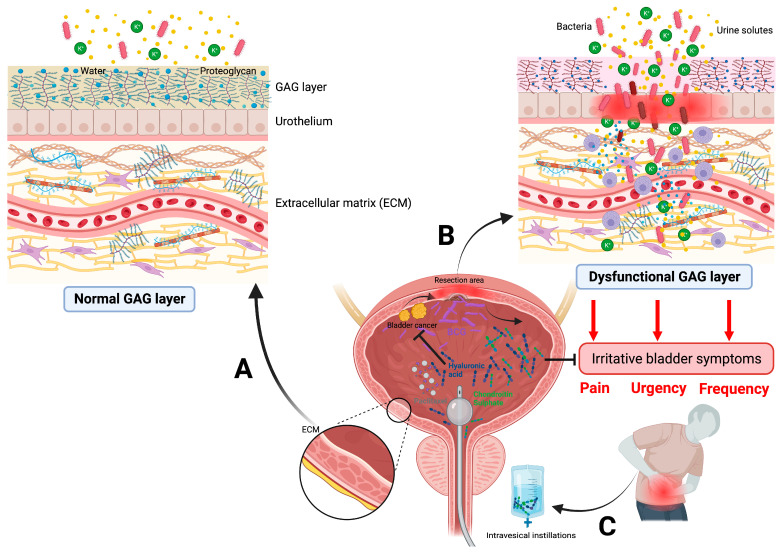
Physiological function and dysregulation of the glycosaminglycan (GAG) layer. (**A**) In physiological conditions, the protective bladder wall lining, consisting of GAGs including hyaluronic acid, chondroitin sulfate, heparin sulfate, and keratin sulfate, protects the bladder and prevents the adhesion of urine solutes and bacteria. (**B**) Damage to this GAG layer is triggered not only by bladder infections but also by bladder tumor surgery or intravesical instillation treatments. As a consequence, the penetration of urine molecules into the bladder tissue results in an inflammatory process, which leads to the activation of mast cells. Then, cytokines, histamine, proteolytic enzymes, and vasoactive amines are released, resulting in a pro-inflammatory response in the urothelium resulting in extracellular matrix degradation. Patients complain of bladder pain, urgency, and frequency. (**C**) Intravesical instillations of hyaluronic acid and chondroitin serve to re-establish the epithelial integrity. The initial data also suggest an antitumor effect of hyaluronic acid in combination with paclitaxel in BCG-refractory tumors [19,20].

The GAG layer is a protective mucus layer over the urothelium with a specific permeability, which protects the bladder wall as a physical barrier. When the GAG layer is depleted during bladder infections, interstitial cystitis, transurethral bladder cancer resection, or intravesical treatments with BCG, the GAG deficiency results in a “leaky” epithelium, allowing the passage and influx of potassium ions, bacteria, and urine solutes towards the bladder tissue, initiating an inflammatory reaction in the bladder wall, thus promoting bladder symptoms such as urgency, frequency, and pain. Since the GAG layer mainly consists of hyaluronic acid, chondroitin sulfate, keratin sulfate, and heparin sulfate, reconstructing the dysfunctional GAG layer by instilling components of the GAG layer into the bladder appears to be a promising therapeutic concept (Figure 2).

Mechanistically, hyaluronic acid can restore the bladder GAG layer by altering epithelial permeability through stimulating the expression of tight junction proteins and by inducing anti-inflammatory mechanisms including the inhibition of mast cell activation and immune cell infiltration [13]. Analyses about the function of specific sulfated GAGs in the bladder has shown that endogenously produced chondroitin sulfate is the dominant sulfated GAG on the urothelial luminal surface, underlining its importance in the bladder barrier function [14]. Therefore, combined instillations of hyaluronic acid and chondroitin (iAluRil) seem to be a reasonable novel therapeutic approach in different bladder diseases resulting from urothelial GAG loss. One instillation consists of 40 mL of sodium hyaluronic 1.6% and chondroitin sulfate 2% in normal saline. iAluRil is applicated intravesically once a week for 8 weeks, then as further needed. Focusing on interstitial cystitis, the first small case series has already shown a good therapeutic efficacy of combined chondroitin and hyaluronic acid instillations (iAluRil) with a significant improvement in bladder symptoms and improved long-term outcomes over a 3-year period [15,16,17].

In BCG-treated bladder cancer patients, instillations of hyaluronic acid can serve two purposes: (a) to restore the physical barrier and to prevent irritative bladder symptoms, and (b) to increase antitumor activity in BCG-refractory bladder tumors, when hyaluronic acid is combined with paclitaxel in therapeutic bioconjugates (ONCOFID-P-B) [18]. ONCOFID-P-B instillations confirm a homogeneous distribution over the whole internal surface of the bladder mucosa with a low or absent systemic absorption resulting in low toxicity rates [18]. In addition, ONCOFID-P-B applied by intravesical instillations in BCG-refractory carcinoma in situ (CIS) showed very promising results in a phase I study [19]. A follow-up trial by Hurle et al. confirmed a high rate of complete responders (CR) in 75% of patients when administered weekly for up to 12 consecutive weeks, maintaining CR in 40% of patients after 15 months of follow-up [20].

Focusing on BCG-induced cystitis, Imperatore et al. reported a case series of 20 patients with refractory BCG-induced cystitis showing a significant and durable improvement of bladder pain, urgency, and frequency through bladder instillations of chondroitin and hyaluronic acid [21]. In detail, the mean (SD) visual analogue scale (VAS) score for urinary urgency and bladder pain decreased from 7.8 (0.5) and 7.2 (1.0) at baseline to 4.7 (1.1) and 4.2 (0.9) at the end of the intravesical chondroitin and hyaluronic acid instillations. All outcome measures remained stable at 6 months and at a 1-year follow-up [21]. However, all studies on the treatment of BCG-related cystitis included only a small number of patients with a high heterogeneity concerning follow-up, BCG strains, BCG schedules, and clinical endpoints.

## 4. Methods

After approval by the local ethics committee (study number 1006/2017), the medical records of patients with primary, high-risk NMIBC and consecutive intravesical BCG therapy after a transurethral resection of the bladder (TURB) were reviewed retrospectively. BCG-induced cystitis was defined as persistent irritative bladder symptoms with repetitive negative urine cultures. A second TURB within 3–6 weeks after the initial resection was performed in all patients to exclude a residual tumor or understaging. According to the EAU guidelines [2], intravesical BCG induction therapy was given in a 6-weekly schedule once a week, followed by maintenance therapy for 1–3 years (3-weekly once a week at 3, 6, 9, 12, 18, 24, 30, and 36 months). Each intravesical instillation through a sterile disposable catheter contained 2 × 10^8^ to 3 × 10^9^ viable units from a live attenuated strain of BCG bacteria seed RIVM derived from seed 1173-P2 (BCG Medac, Wedel, Germany). Each solution was retained up to 1–2 h. The BCG-induced cystitis was treated with combined intravesical chondroitin and hyaluronic acid instillations. Evaluating the frequency, severity, and impact on the QoL of patients, two questionnaires—the Incontinence Questionnaire—Urinary Incontinence Short Form (ICIQ-UI SF) and the Incontinence Quality of Life questionnaire (I-QoL)—were collected dynamically at different time points (at baseline and at 6, 12, and 24 weeks) during/after chondroitin and hyaluronic acid instillations. Oncologic surveillance was performed with a cystoscopy and a urinary (voided urine and bladder washing) cytology every 3 months for a period of 2 years, then every 6 months for 5 years, and once every year thereafter [2]. Computed tomography urography was performed initially at the time of diagnosis and repeated once a year. A muscle-invasive bladder cancer detected during a follow-up (progression) or a high-grade relapse after the BCG therapy was defined as BCG failure.

## 5. Results

**Figure 3:**
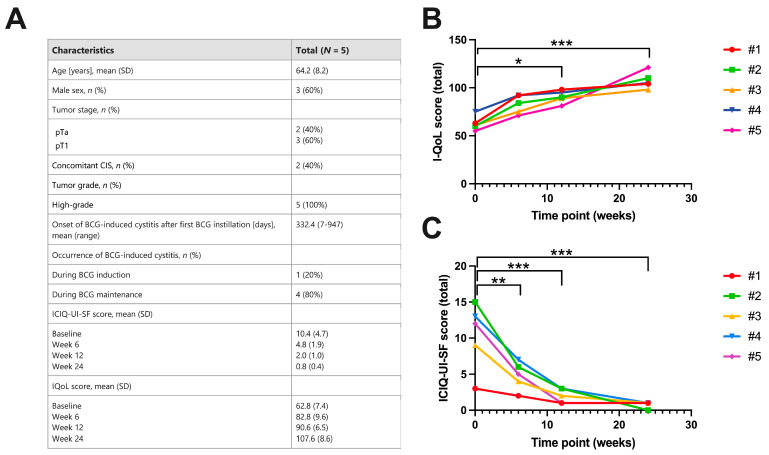
Retrospective case series of five patients with an overview of (**A**) the descriptive and histopathological characteristics, as well as (**B**,**C**) questionnaire outcomes. Both the I-QoL (**B**) and ICIQ-UI-SF (**C**) questionnaires were evaluated at baseline and at 6, 12, and 24 weeks after the start of chondroitin and hyaluronic acid instillation therapy in patients with persistent BCG-induced cystitis. * *p* < 0.05; ** *p* < 0.01; and *** *p* < 0.001; repeated measures ANOVA.

Here, we provide a further case series of five patients with refractory BCG-induced cystitis treated with combined hyaluronic acid and chondroitin instillations. iAluRil was instillated once a week for 6 weeks. The mean (range) age was 64.2 (56–75) years. Three (60%) of the five patients were male. All patients were defined as BCG responders with no tumor recurrence during a mean oncological follow-up of 36.5 months. All patients confirmed a primary high-risk NMIBC. In four of the five patients, the BCG-induced cystitis occurred during the BCG maintenance therapy. In detail, the mean (range) onset of irritative bladder pain symptoms was 332.4 (7–947) days after starting the intravesical BCG treatment (Figure 3A). In all patients, a significant improvement of incontinence and irritative symptoms was already noticed 6 weeks after the beginning of chondroitin and hyaluronic acid instillations. Focusing on QoL, the mean (SD) IQoL score was improved from 62.8 (7.4) at baseline to 82.8 (9.6) at 6 weeks (Figure 3B). The mean (SD) ICIQ-UI-SF score decreased from 10.4 (4.7) at baseline to 4.8 (1.9) at 6 weeks after starting the combined chondroitin and hyaluronic acid instillations (Figure 3C). A detailed overview of the descriptive characteristics and questionnaire outcomes are presented in Figure 3.

## 6. Limitations

One of the major limitations of the present case series is the heterogeneous and retrospective character of the limited patient number, which restricts the statistical methods of interpretation. Therefore, further prospective and multi-institutional randomized trials with sufficient statistical power and long-term follow-ups are required to validate these preliminary findings.

## 7. Conclusions and Future Perspectives

In summary, different inflammatory bladder diseases, including BCG-induced cystitis, are linked in the first step of the disease to the deficiency of the mucous layer of the GAG, with a limited ability to repair the damaged urothelial layer and to restore the GAG. The early repair of the GAG layer can be supported by the intravesical application of the components of the GAG, such as hyaluronic acid and chondroitin sulfate, preventing chronic bladder inflammation. Further prospective randomized studies with larger numbers of cases are needed to confirm this very promising preliminary data.

## Data Availability

The data presented in this study are available on request from the corresponding author.
